# Intrinsisch photosensitive retinale Ganglienzellen

**DOI:** 10.1007/s00347-021-01476-4

**Published:** 2021-08-04

**Authors:** Leonie Kinder, Teele Palumaa, Moritz Lindner

**Affiliations:** 1 Institut für Physiologie und Pathophysiologie, Philipps-Universität Marburg, Deutschhausstr. 2, 35037 Marburg, Deutschland; 2 Nuffield Department of Clinical Neuroscience, Universität Oxford, Oxford, Großbritannien; 3 East Tallinn Central Hospital Eye Clinic, Tallinn, Estland

**Keywords:** Melanopsin, Intrinsisch photosensitive retinale Ganglienzellen, Zirkadianer Rhythmus, Pupillenlichtreflex, Nicht-bildformendes Sehen, Optogenetik, Melanopsin, Intrinsically photosensitive retinal ganglion cells, Circadian rhythms, Pupillary light reflex, Non-image-forming vision, Optogenetics

## Abstract

**Hintergrund:**

Melanopsin exprimierende, intrinsisch-photosensitive retinale Ganglienzellen (ipRGCs) bilden neben Stäbchen und Zapfen die dritte Klasse von retinalen Photorezeptoren. Diese kleine, heterogene Zellfamilie vermittelt ein weites Spektrum an Aufgaben überwiegend des nicht-bildformenden Sehens.

**Fragestellung:**

Diese Arbeit soll einen Einblick in das aktuelle Verständnis der Funktion und der funktionellen Diversität der ipRGCs geben sowie klinisch und translational relevante Aspekte beleuchten.

**Material und Methoden:**

Narrative Übersichtsarbeit.

**Ergebnisse:**

ipRGCs machen etwa 1–2 % aller retinalen Ganglienzellen aus und bilden dabei 6 spezialisierte Subtypen. Mit ihrem Photopigment Melanopsin sind sie in der Lage, unabhängig von synaptischem Input Lichtinformationen an das Gehirn weiterzuleiten oder lichtabhängig zu modifizieren. Je nach Subtyp vermitteln sie so nichtvisuelle Aufgaben wie die Synchronisation der inneren Uhr oder den Pupillenreflex, greifen aber auch in das bildformende System ein. ipRGCs weisen eine differenzielle Widerstandskraft gegenüber Optikusschädigung auf, was sie zu einem attraktiven Studienobjekt für die Entwicklung neuroprotektiver Therapieansätze macht. Melanopsin rückt zudem als optogenetisches Werkzeug, etwa in der prosthetischen Gentherapie, in den Fokus.

**Schlussfolgerungen:**

Häufige klinische Beobachtungen lassen sich nur mit Kenntnis des ipRGC-Systems verstehen. Ihre neuronale Vernetzung und die intrazelluläre Signalverarbeitung sind Gegenstand aktiver Forschung, die neue translationale Ansätze hervorbringt.

## Abstract (en)

**Background:**

Melanopsin-expressing intrinsically photosensitive retinal ganglion cells (ipRGC) area third class of photoreceptors in the retina in addition to rods and cones. They are a small heterogeneous population of cells primarily mediating non-image-forming visual functions.

**Objective:**

This article provides an overview of the current understanding of the functions and the diversity of ipRGCs. It moreover gives an insight into clinically and translationally relevant aspects and treatment options.

**Material and methods:**

Narrative review article.

**Results:**

ipRGCs make up ~1–2% of all retinal ganglion cells and are divided into 6 specialized subtypes. With the photopigment melanopsin they can trigger light responses without rod or cone input and can relay irradiance information to various centers of the brain. Depending on the subtype, ipRGCs mediate non-image-forming tasks, such as the pupillary light reflex or synchronizing the circadian clock, and image-forming tasks, such as contrast optimization. ipRGCs exhibit differential resilience against optic nerve damage, making them an interesting study object for the development of neuroprotective strategies. In addition, melanopsin is an attractive optogenetic tool for vision restoration.

**Conclusion:**

Knowledge on ipRGC physiology is indispensable for understanding frequent clinical observations. Their functional and morphological features are the subject of active research, which highlights novel translational strategies.

Das menschliche Auge dient 2 sehr unterschiedlichen sensorischen Aufgaben. Einerseits nehmen wir über das Auge Lichtreize wahr, die in der Retina und im Gehirn verarbeitet werden, um uns so mit räumlichen und zeitlichen Informationen über unsere Umgebung zu versorgen. Während dieses sog. *bildformende Sehen* unser Bewusstsein erreicht, laufen Prozesse des *nicht-bildformenden* (nbf) Sehens ab, ohne, dass wir uns ihrer bewusst werden. Das *nbf*-Sehen dient überwiegend dazu, Körperfunktionen langfristig an die Lichtverhältnisse der Umgebung anzupassen.

Beispiele für das nbf-Sehen sind etwa die Synchronisation der inneren Uhr mit dem Tagesrhythmus aus Hell und Dunkel, der Pupillenreflex oder die lichtabhängigen Effekte etwa auf Stimmung und Stoffwechsel. Die Ansprüche an bildformendes und nbf-Sehen sind höchst unterschiedlich: Um einem Hindernis ausweichen zu können, bedarf es eines räumlich und zeitlich hochaufgelösten bildformenden Sehens. Die innere Uhr sollte hingegen von einem sehr kurzen oder fokalen Ereignis – etwa dem Blitz bei einem Gewitter – nicht beeinflusst werden, sodass das nbf-Sehen räumlich und zeitlich integrieren muss (Abb. 1a). Wie das Auge diesen beiden so unterschiedlichen Aufgaben gerecht werden kann, war lange weitgehend unklar.

**Figure 1:**
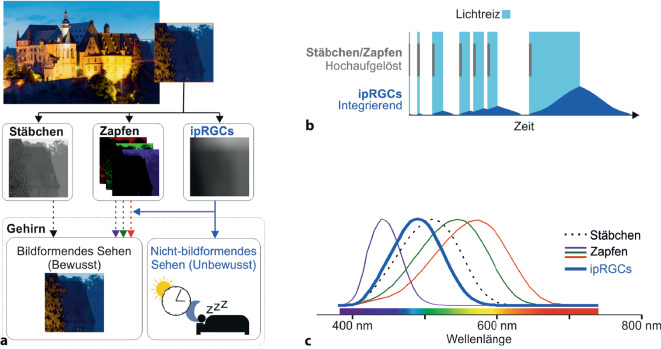

In den 90er-Jahren beobachteten Czeisler et al. an blinden Probanden, dass sich durch Lichtreize die Produktion des zirkadian ausgeschütteten Melatonins unterdrücken ließ [8]. Während diese Arbeit noch Raum für alternative Erklärungsansätze ließ, lieferten wenig später tierexperimentelle Untersuchungen Klarheit: Stäbchen- und zapfenlose Mäuse waren in der Lage, ihre innere Uhr mit äußeren Lichtverhältnissen zu synchronisieren. Da diese Fähigkeit jedoch nach bilateraler Enukleation verloren ging, musste neben Stäbchen und Zapfen eine dritte Art von Photorezeptoren im Auge existieren [13].

Etwa zeitgleich wurde mit Melanopsin ein neues Mitglied der Opsinfamilie identifiziert und in ungefähr 2 % der retinalen Ganglienzellen (RGCs) nachgewiesen [4]. Diese Melanopsin-positiven RGCs waren auch ohne Signale von Stäbchen oder Zapfen in der Lage, Lichtantworten zu generieren, weshalb sie den Namen *intrinsisch photosensitive retinale Ganglienzellen* (ipRGCs) erhielten. Sie zeigten Melanopsin-vermittelt träge, lang anhaltende und kaum adaptierende Lichtantworten, deren spektrales Maximum im kurzwelligen Bereich lag (Abb. 1b, c). Diese spektralen und kinetischen Charakteristika entsprachen sehr gut vorrangegangenen Beobachtungen der inneren Uhr [4]. In der Tat ließ sich nach genetischer Ablation von Melanopsin in bereits stäbchen- und zapfenlosen Mäusen keinerlei Einfluss von Licht auf die innere Uhr mehr nachweisen [17]. Gerade das lang anhaltende Antwortverhalten des Melanopsin stellte sich als funktionell sehr bedeutend heraus: Im Gegensatz zu Stäbchen und Zapfen ermöglicht es ipRGCs, anstelle von Kontrasten auch Informationen über die absolute Helligkeit zu vermitteln [10].

## ipRGCs: eine heterogene Zellpopulation

**Figure 2:**
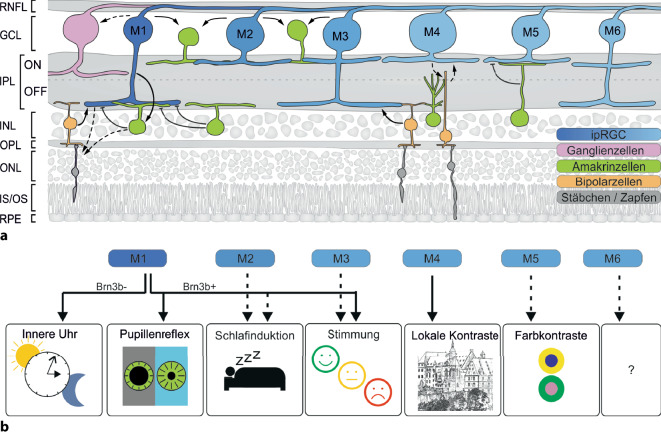

Bereits in den ersten Untersuchungen zeigte sich, dass es sich bei ipRGCs keineswegs um eine homogene Zellpopulation handelt [16]. Anhand morphologischer Kriterien werden inzwischen 6 (murine) ipRGC-Subtypen (M1–M6) unterschieden [1]. So kennzeichnet die zuerst entdeckten M1-Zellen ein großer, aber wenig verzweigter Dendritenbaum, der ausschließlich in der OFF-Sublamina der inneren plexiformen Schicht (IPL) stratifiziert. Im Gegensatz dazu stratifizieren alle anderen ipRGC-Subtypen nur (bzw. im Falle von M3 und M6: auch) in der ON-Sublamina. Von M2 zu M6 nimmt zudem die Komplexität des Dendritenbaums stark zu (Abb. 2a und 3; [35]).

**Figure 3:**
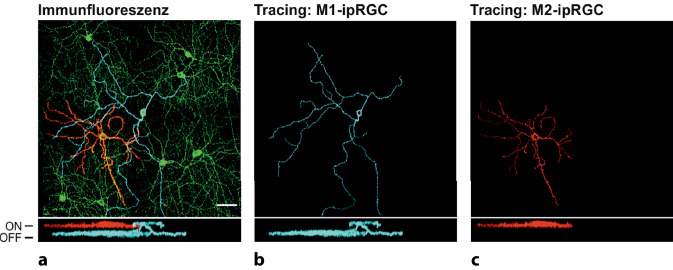

**Figure 4:**
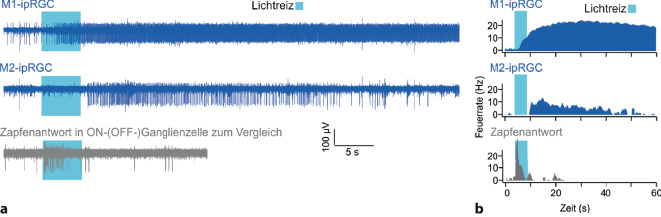

Die Unterschiede zwischen den ipRGCs beschränken sich allerdings nicht nur auf die Morphologie. Besonders deutlich wird dies für die M1-Zellen: Anhand der Expression des Transkriptionsfaktors Brn3b lassen sich M1-Zellen nochmals in 2 Subgruppen mit klar getrennten Aufgaben (Abb. 2b und s. unten) unterteilen: Während bei nahezu allen ipRGCs dieser Brn3b nachgewiesen werden kann, ist dies bei einer kleinen Subpopulation von M1-Zellen nicht der Fall. Von funktioneller Bedeutung ist auch, dass sie Melanopsin sehr unterschiedlich stark exprimieren und verschiedene Signaltransduktionskaskaden nutzen. So ist ihr intrinsisches Antwortverhalten auf Lichtreize sehr heterogen: M1-Zellen reagieren sensitiv, schnell und lang anhaltend, während M2–M6-Zellen (mit wiederum erheblicher Variabilität) wenig sensitiv, langsam und transient reagieren (Abb. 4; [1]). Auch die Art, wie sie intrinsische Lichtinformationen mit extrinsischen, d. h. von Stäbchen oder Zapfen zugeleiteten Informationen, integrieren, ist höchst unterschiedlich [35].

Inzwischen wurde für ipRGCs ein ganzes Potpourri an zentralen Projektionsgebieten identifiziert, die sich wiederum zwischen den einzelnen Subtypen stark unterscheiden. Diese Vielzahl an Projektionsgebieten hat Forscher dazu bewogen, mögliche Aufgaben der ipRGCs sehr breit zu untersuchen. Vieles ist diesbezüglich noch Gegenstand andauernder Diskussion. Konsens besteht im Wesentlichen zu M1- und M4-Zellen (Abb. 2b). Klar ist aber, dass es die Diversität in Projektionsmustern, Konnektivität und biophysikalischen Eigenschaften ist, die es ipRGCs ermöglicht, spezifisch ihren physiologischen Aufgaben nachzukommen. In den folgenden Abschnitten werden daher die wichtigsten Aufgaben der ipRGCs dargestellt und – soweit bekannt – die Rollen der einzelnen Subtypen erläutert. Unser aktuelles Wissen beruht dabei zu einem großen Teil auf Untersuchungen an Mäusen. Verfügbare Daten zeigen aber, dass die ipRGCs von Affen und Menschen insgesamt den Murinen sehr ähneln [10].

## Synchronisation der inneren Uhr und Schlaf

Fast alle Lebewesen besitzen innere Uhren, die ihnen ermöglichen, periodische Veränderungen in ihrer Umgebung zu antizipieren. Die prominenteste dieser Veränderungen ist der immer wiederkehrende Wechsel aus Tag und Nacht. Diese fundamentale Aufgabe wird exklusiv von einer Subgruppe von M1-Zellen vermittelt, die sich von allen anderen ipRCGs durch fehlende Expression von Brn3b abhebt. Aufgrund dieser Eigenschaft lassen sich M1^Brn3b−^ sehr einfach getrennt von den anderen ipRCG-Subtypen untersuchen. Die Axone dieser M1^Brn3b−^ ziehen praktisch ausschließlich in den Nucleus suprachiasmaticus (SCN) des Hypothalamus, dem Sitz der zentralen inneren Uhr. Durch Ablation speziell dieser Subpopulation geht die Fähigkeit, die innere Uhr an äußere Lichtverhältnisse anzugleichen, verloren ([14, 23]; Abb. 2b, *links*).

Neben der inneren Uhr vermitteln ipRGCs auch unmittelbare Effekte auf Schlaf. Akute Blaulichtexposition induziert im Mausmodell eine sofortige Weckreaktion. Beobachtungen legen nahe, dass dies über Stimulation eines ipRGC-Subtyps mit starker intrinsischer (d. h. Melanopsin-vermittelter) Lichtantwort, a. e. den M1-Zellen über Projektionen in den SCN und nichtvisueller Anteile des Corpus geniculatum laterale (CGL) gelingt [23, 28]. Konsistent mit dieser Beobachtung lässt sich bei humanen Probanden die subjektiv empfundene Müdigkeit durch Melanopsin stimulierendes Blaulicht reduzieren, während dies durch langwelligeres Licht nicht der Fall ist [5].

Bemerkenswerterweise können ipRGCs – zumindest bei Mäusen – ebenfalls zur Schlafinduktion beitragen [26]. Interessant ist die Beobachtung, weil sie die Frage aufwirft, wie ipRGCs diese beiden zueinander gegensätzlichen Aufgaben wahrnehmen können. Die spektrale Zusammensetzung des Lichtreizes ist ausschlaggebend: Während Licht mit hohem kurzwelligem Anteil die oben beschriebene Weckreaktion einleitet, induziert Licht mit höherem langwelligen Anteil Schlaf, indem es bevorzugt ipRGCs mit schwächerer intrinsischer und stärkerer extrinsischer Signalantwort (also Zellen vom Nicht-M1-Subtyp) aktiviert [28]. Um welchen Subtyp es sich genau handelt, ist noch umstritten [28, 33]. Von M1–M3-Zellen ist beschrieben, dass sie in Kerngebiete projizieren (Nucleus praeopticus ventrolateralis und Colliculi superiores), die sie als Vermittler der Schlafinduktion infrage kommen lassen ([10]; Abb. 2b).

## Pupillenreflex

Über die Regulation des Pupillendurchmessers setzt der Pupillenreflex Sensitivität und Auflösungsvermögen des optischen Systems in eine dynamische Beziehung und maximiert so den Informationsfluss in Abhängigkeit des verfügbaren Lichts. Durch chemische Ablation der ipRGCs lässt sich im Mausmodell der Pupillenreflex fast komplett zum Erliegen bringen [14]. In einem eleganten Versuch konnte durch Vorderkammerinjektion von Fluoreszenzmarker-kodierenden Pseudorabiesviren der komplette Reflexbogen dargestellt werden [2]: Motorneurone des Ncl. Edinger-Westphal werden aus der Schalenregion des Nucleus olivaris pretectalis (OPN) versorgt, der seine retinalen Afferenzen ausschließlich von ipRGCs bezieht, die sich anhand ihrer Morphologie als M1- (genauer, M1^Brn3b+^-)Zellen identifizieren ließen ([2, 23]; Abb. 2a, b).

Bedeutend für den intakten Pupillenreflex ist das Zusammenspiel aus Melanopsin-vermittelter intrinsischer Photosensitivität und extrinsischen Signalen. ipRCGs erhalten Signale aus Stäbchen und Zapfen (Abb. 2a, *Pfeile*) und vermitteln so, fast ohne relevanten Beitrag von Melanopsin, die initiale, schnelle und kräftige Pupillenkontraktion [14]. Diese ist ohne Melanopsin-Input aber nur sehr transient. Eine langfristige Kontraktion, etwa bei Sonnenaufgang zur Anpassung an das einbrechende Tageslicht, findet ohne Melanopsin kaum statt [20]. Diese Tatsache macht man sich zunutze um mittels der sog. postilluminatorischen Pupillenreaktion (PIPR) beim Menschen die Funktion der ipRGCs (genauer: des Melanopsins) zu untersuchen.

## Stimmung und Affekt

Licht hat einen bedeutenden Einfluss auf die Stimmung und ist etwa im Kontext saisonal abhängiger Depressionen (SAD) und deren Therapie von Bedeutung [32]. ipRGCs innervieren mehrere Kerne des limbischen Systems [1], und ein Polymorphismus (p.P10L) im *Melanopsin*-Gen ist mit einem rund 6‑fach erhöhten Risiko einer SAD assoziiert [32]. Diese Beobachtungen stießen eine ganze Reihe weiterer funktioneller Untersuchungen an. So hielten LeGates et al. Labormäuse unter einem beschleunigten Tag‑/Nacht-Zyklus. Dieser sog. T7-Zyklus induziert einen depressiven Phänotyp, ohne die innere Uhr im SCN zu beeinflussen [22]. Vor wenigen Jahren konnten dann Fernandez et al. zeigen, dass dieser Effekt von M1^Brn3b+^- und M3-Zellen vermittelt wird, die in den Nucleus perihabenularis projizieren (Abb. 2b, *Mitte*). Fehlten diese Zellen, so waren die Tiere gegen die T7-Zyklus-induzierte Depressionsentwicklung „immun“ [12].

## Die Rolle von ipRGCs im bildformenden Sehen

Lange Zeit galt den ipRGCs Aufmerksamkeit v. a. im Kontext des nbf-Sehens. Früh gab es jedoch funktionelle und strukturelle Hinweise auf eine Beteiligung auch am bildformenden Sehen [15]. ipRGCs projizieren auch in die dorsalen Anteile des CGL und leiten so Helligkeitsinformationen bis in den Kortex weiter. Die aktuelle Datenlage deutet darauf hin, dass es sich hierbei zumindest überwiegend um M4-Zellen handelt [11]. Diese in den CGL-projizierenden ipRGCs reichen aus, um stäbchen- und zapfenlosen Mäusen sogar das Erkennen grober Muster zu ermöglichen ([1]; Abb. 2b). Umgekehrt macht sich bei Tieren mit intakten Stäbchen und Zapfen das Fehlen von Melanopsin – oder die Ablation von ipRGCs insgesamt – in einer Herabsetzung der Kontrastwahrnehmung bemerkbar [1].

M4-Zellen (auch als α‑ON-Ganglienzellen bekannt) integrieren extrinsische und intrinsische Signale, um *selbst* Kontrastinformation ans Gehirn weiterzuleiten. Dabei wird in M4-Zellen durch Stimulation von Melanopsin das Schließen von Hintergrundkaliumkanälen vermittelt und so lang anhaltend die elektrische Erregbarkeit erhöht. So werden Signale, die sie von Stäbchen und Zapfen erhalten, vereinfacht ans Gehirn weitergeleitet. Bemerkenswerterweise zeigte diese Untersuchung aber auch, dass dieser Signalweg nicht in allen ipRGC-Subtypen gleichermaßen genutzt wird, in M1-Zellen etwa senkt Melanopsin-Aktivierung sogar die elektrische Erregbarkeit [36].

ipRGCs beeinflussen das bildformende Sehen aber nicht nur, indem sie selbst in bildformende Hirnareale projizieren. Sie interagieren auch innerhalb der Netzhaut mit umliegenden Zellen und tragen so *indirekt* zum bildformenden Sehen bei. Über diesen Mechanismus sind auch ipRGC-Subtypen beteiligt, die selbst nicht ins CGL projizieren. So modifizieren sie die Aktionspotenzialfrequenz umliegender Ganglienzellen und erhöhen so den Informationsgehalt der über den N. opticus gesendeten Signale [27]. Die zugrunde liegenden Mechanismen sind nicht vollständig geklärt. Klar ist jedoch, dass Melanopsin die B‑Welle im Zapfen-Elektroretinogramm (ERG) verändert [15], indem M1-Zellen über chemische Synapsen mit dopaminergen Amakrinzellen die Erregbarkeit von Neuronen der äußeren Netzhaut modulieren (Abb. 2a **– ***Pfeile*, [29]). Daneben formen ipRCGs Synapsen mit einer Reihe weiterer Amakrinzelltypen sowie womöglich auch direkt mit Zapfen.

## Aktuelle Entwicklungen

### ipRGCs und Myopie

Die Aufenthaltsdauer im Freien ist eine der Haupteinflussfaktoren für Myopieentwicklung im Kindes- und Jugendalter [19]. Eine gestörte lichtinduzierte Dopaminfreisetzung ist dabei als pathogenetischer Faktor etabliert. Wie genau es dazu kommt, ist jedoch unklar. Eine wachsende Anzahl an Untersuchungen weist nun auf eine Rolle von Melanopsin hin. Dies würde biologisch Sinn ergeben, da ipRGCs (mindestens M1-Zellen) in der Lage sind, die Dopaminausschüttung aus Amakrinzellen zu beeinflussen **(**Abb. 2a**;** [29]). In der Tat sind Mäuse, denen das *Melanopsin*-Gen fehlt, in ihrer Jugend deutlich myoper als ihre Geschwister mit intaktem *Melanopsin*-Gen [6]. Umgekehrt waren Versuchstiere, wenn sie unter kurzwelligen (Melanopsin stimulierenden) Lichtbedingungen aufwuchsen, deutlich weniger myop, als wenn sie unter langwelligen Lichtbedingungen lebten [38]. Humane Daten hierzu sind leider bisher nur begrenzt verfügbar und widersprüchlich [20, 21]. Beide Studien untersuchen eine Assoziation zwischen Melanopsin-vermitteltem PIPR und Myopie. Da mittels des PIPR am ehesten nur ein bestimmter ipRGC-Subtyp getestet wird, ist jedoch fraglich, inwieweit der PIPR als Messgröße hier geeignet ist.

### Optikuserkrankungen und ipRGCs

Versuche an Nagetieren zeigen, dass ipRGCs im Vergleich zu anderen RGC eine größere Resistenz gegenüber mechanischer Optikusschädigung und toxischen Einflüssen aufweisen [18, 31]. Sehr früh ergaben sich Hinweise, dass eine ähnliche Resistenz auch gegenüber Glaukomschäden bestehen könnte [24]. Zwar konnten Folgestudien diese Beobachtung nicht vollständig bestätigen (s. unten) [18], sie führten dennoch dazu, dass die Rolle von ipRGCs bei Optikuserkrankungen auch beim Menschen untersucht wird.

Inzwischen existiert eine ganze Reihe von Studien, die die Widerstandskraft von ipRGCs bei Glaukom mittels PIPR untersuchen. Trotz Unterschieden im Detail ist das einhellige Fazit dieser Studien, dass ipRGCs bei Glaukom ebenfalls zugrunde gehen. Dabei zeigt sich eine Korrelation des PIPR sowohl mit perimetrischen als auch klinisch-morphologischen Befunden [21]. Eine relative Widerstandskraft kann hier nicht ausgeschlossen werden – „immun“ gegen Glaukomschaden sind ipRGCs beim Menschen jedoch keineswegs. Störungen des Schlaf- und Tag-Nacht-Rhythmus sind bei Glaukomerkrankungen häufig [30]. Es ist also gut vorstellbar, dass der Verlust der ipRGCs eine wichtige Rolle spielt. Ein ganz anderes Bild bietet sich bei hereditären Optikuserkrankungen. Sowohl bei Leberscher hereditärer Optikusatrophie als auch bei OPA1-assoziierter Optikusatrophie bleiben ipRGCs über den gesamten Krankheitsverlauf erhalten [21].

In der Hoffnung, neuroprotektive Mechanismen zu identifizieren, welche sich auch auf andere Zellen übertragen lassen, werden die Ursachen dieser Resistenz vermehrt erforscht [7]. Eine differenzielle Expression von NMDA-Rezeptoren oder des Neuropeptids PACAP sind in Diskussion – insgesamt sind die Vorgänge auf molekularer Ebene aber kaum verstanden [7]. Neue Methoden wie die Einzelzelltranskriptomik könnten hier zeitnah neue Ansatzpunkte bieten.

### Melanopsin-basierte Ansätze in der Gentherapie

Durch Einbringen lichtsensitiver Proteine mittels gentherapeutischer Ansätze lassen sich Lichtantworten auch in Zellen hervorrufen, die nativ nicht selbst auf Lichtreize reagieren („Optogenetik“). Ein attraktives Anwendungsfeld für einen solchen Ansatz sind etwa terminale Stadien degenerativer Netzhauterkrankungen: Durch Expression lichtsensitiver Proteine etwa in Bipolar- oder Ganglienzellen lässt sich im Tiermodell bildformendes Sehen auch nach vollständiger Degeneration von Stäbchen und Zapfen wiederherstellen. Klinische Studien laufen (ClinicalTrials.gov: NCT02556736, NCT03326336), und vielversprechende erste Ergebnisse wurden soeben publiziert: Ein Patient, der zuvor nur Lichtscheinwahrnehmung besaß, war mit seinem behandelten Auge und eines in eine Brille eingebauten Projektors in der Lage, verschiedene Objekte im Nahbereich zu lokalisieren und zu zählen [34]. Als natives Säugetierprotein, das in der Lage ist, eine ubiquitäre Signalkaskade auszulösen, ohne auf Retinal-Recycling durch das retinale Pigmentepithel angewiesen zu sein, gilt Melanopsin hier ein besonderes Interesse [9, 25]. Aber auch jenseits der Augenheilkunde werden analoge Anwendungsfelder für Melanopsin erforscht: etwa als optisch steuerbarer kardialer oder neuronaler Schrittmacher [3, 37].

## Fazit für die Praxis

- ipRGCs sind die dritte Klasse von Photorezeptoren in der Netzhaut.
- Sie sind essenziell für grundlegende Aufgaben wie der Synchronisation der inneren Uhr, dem Pupillenreflex oder der Lichteinflüsse auf Stimmung.
- Ein Verlust der ipRGCs (z. B. durch bilaterale Enukleation) führt zu weitreichenden Störungen von Schlafrhythmus und Psyche.
- Aktuelle Entwicklungen weisen auf eine Rolle der ipRGCs bei der Entstehung der Myopie hin.
